# Assessing the nutritional value and health risks of special low‑protein foods: narrative review

**DOI:** 10.1186/s13023-026-04266-w

**Published:** 2026-03-06

**Authors:** Maryam Ziadlou, Anita MacDonald

**Affiliations:** 1 Department of Food Science and Technology, Science and Research Branch, Islamic Azad University, Tehran, Iran; 2 Dietetic Department, Birmingham Children’s Hospital, Steelhouse Lane, Birmingham, B4 6NH UK

**Keywords:** Amino acids disorders, Low protein diet, Special low protein foods, Food additives, Gut microbiota, Bioactive components

## Abstract

Special low-protein foods (SLPFs) are essential for patients with disorders of inherited amino acid metabolism that require lifelong dietary protein restriction to prevent severe neurocognitive effects and even death. Conditions such as phenylketonuria (PKU), tyrosinemia (TYR), maple syrup urine disease (MSUD), homocystinuria (HCU), and urea cycle disorders (UCD) depend on these foods to support metabolic control and dietary adherence. SLPFs provide satiety, energy, and help prevent catabolism, but their nutritional composition poses challenges. Most SLPFs are formulated using isolated starches as the primary macronutrient base. Hydrocolloid fibers are commonly added to improve texture, consistency, shelf life, and water or gas retention. These ingredients form the backbone of SLPFs production and are consistently used across different regions worldwide, reflecting a standardized approach to their formulation. However, their potential adverse effects include suppression of gut microbiota, gut dysbiosis, increased inflammatory markers, overweight, and obesity, all of which raise cardio‑metabolic risks. Strengthening the nutritional quality of SLPFs through natural plant sources may help mitigate their potential adverse outcomes while ensuring patients’ dietary needs are met. Therefore, it is important to explore natural low‑protein alternatives that can both support sustainable food production and promote long‑term health benefits.

## Background

Foods for Special Medical Purposes (FSMPs) are specialized nutritional products developed to meet the distinct dietary needs of individuals with medically prescribed dietary restrictions [1]. These formulations are designed to be low, or completely free, from certain ingredients or nutrients, such as sucrose, lactose, galactose, fructose, gluten, cow’s milk protein, or specific amino acids, that could otherwise compromise health in the context of specific medical conditions. Amino acid metabolism disorders (AMDs), including phenylketonuria (PKU), tyrosinemia (TYR), homocystinuria (HCU), and maple syrup urine disease (MSUD), necessitate the use of amino acid-based formulas specifically adapted to compensate for impaired metabolic pathways [2–5]. Due to the inability to metabolize one or more amino acids, affected individuals must adhere to lifelong restrictions of natural protein intake. Nutritional management therefore relies on supplementation with special low-protein foods (SLPFs), for example, low-protein bread and flour, to ensure adequate energy intake [4, 6–9]. However, their global availability remains limited, with access frequently constrained by inadequate insurance coverage. Furthermore, there are currently no specific legislative frameworks or regulatory provisions governing the nutritional composition of SLPFs [8, 10–12], resulting in variability in quality and nutritional adequacy across regions.

In these conditions, strict adherence to dietary treatment is essential to prevent severe complications. Without appropriate management, the accumulation of amino acids or their toxic metabolites can result in brain toxicity, intellectual disability, progressive neurological impairment, and, in extreme cases, death [5, 13–18]. This narrative review aims to describe the benefits of SLPFs, while also addressing the health burdens associated with their use in AMD’s. It provides new insights into their nutritional composition and highlights the need to improve ingredient quality for better long-term health outcomes.

### Special-low-protein-foods

SLPFs play a critical role in supporting adherence to protein‑restricted diets by providing satiety and energy, helping to prevent catabolism, and improving overall metabolic control. Sustained access to nutritionally improved SLPFs is therefore essential for maintaining long‑term health outcomes in AMDs [7, 12, 19–22]. They are categorized in 4 groups. See Table 1.

**Table 1:** SLPFs categories

Low protein cereals	Baby cereals, bread, flour, pasta, pancakes, biscuits, cakes and cookies [12, 20,21]
Meat and egg substitutes	Meat, fish, sausage, burgers, and egg replacements [20, 21].
Dairy substitutes	Creams, ice cream, yogurt, milk and cheese replacements [12, 20, 21]
Desserts and savory snacks	Jell, chocolate, fruit bars, crisps and crackers [21].

### Nutritional composition of SLPFs and nutritional profile of ingredients added to SLPFs

Globally, SLPFs are typically formulated from a common set of ingredients. These include starches, sugar, salt, emulsifiers, stabilizers, thickening agents, oils, fibers, and artificial flavors and colors, with variations depending on the specific food category. This standardized reliance on similar components reflects the limited range of available low‑protein raw materials [21]. Studies summarizing the nutritional composition of SLPFs are given in Table 2.

**Table 2:** Studies describing the nutritional composition of SLPFs

Titles	Main conclusions	Author, Year Country
Special low protein foods for PKU: availability in Europe and an examination of their nutritional profile	• SLPFs accessibility issues in Europe and Turkey. • Inadequate details about nutritional composition of SLPFs. • Vitamins and mineral content of most SLPFs were unclear.	Pena, 2015 Portugal [12]
Special low protein foods in the UK: An examination of their macronutrient composition in comparison to regular foods	• Nearly all SLPFs (*n* = 146) have protein content ≤ 1.7 g / 100 g • Over 70% of SLPFs (*n* = 105/146) contained corn starch as one of the isolated starches. • Hydrocolloids (MC, HPMC, cellulose and locust bean gum) were predominantly used as fiber sources. • Palm oil and hydrogenated vegetable oil were key fat sources in SLPFs. • 72% of SLPFs contained added sugar, including milk, fish and meat replacements.	Wood, 2020 UK [21]
Special low protein foods for PKU in Turkey: An examination of their nutritional composition compared to regular foods	• Salt content of all SLPFs was significantly higher than regular products. • Carbohydrate content of most SLPFs in Turkey was high. • Vitamins and mineral content of most of SLPFs were unclear	Arslan,2023 Turkey [19]

#### Protein

SLPFs are specifically formulated to contain very low amounts of protein. In a UK study, the protein content of 100% of SLPFs were below 1.7 g/100 g and 84% had a protein content less than ≤ 0.5 g /100 g (*n* = 122/146) [21]. Pena et al., showed that the protein content of low protein cereals, bread, flour, cookies and milk replacers was below 1.3 g/100g [12].

#### Sugar

A substantial proportion of SLPFs contain added sugar. Wood et al. reported that 72% of products (105/146) included added sugars. Among these, 38% had higher sugar levels than regular foods, with the most significant increases found in burgers, pizza bases, flour, and fish substitutes, where sugar content ranged from 83% to 1000% more. The primary sources included sugar, glucose, and maltodextrin [21]. Furthermore, a study by Garcia et al. indicated that the sugar content in meat and fish substitutes was three to 10 times higher compared to regular foods [23].

#### Starch/carbohydrate

Because grain-derived flours are unsuitable for SLPFs due to their protein content, starches serve as the primary ingredients in these formulations. Maize and potato starches are most frequently employed, reflecting their natural low protein content. In a survey of commercially available SLPFs, Wood et al. reported that over 70% (105/146) contained maize/corn starch, while 56% (82/146) incorporated potato starch [21]. Similarly, Garcia reported that 67% of SLPFs have a higher carbohydrate content, primarily from isolated starches, compared to regular foods. They found that meat substitutes contained 20 times more carbohydrate than their conventional equivalents, while cheese substitutes contained 18 times more [23]. Incorporating resistant starches and dietary fibres may offer viable alternatives.

#### Oil/fat

Wood et al. indicated that hydrogenated vegetable oils and palm oil were the most frequently used fats in SLPFs, with 80% of low-protein snacks containing palm oil compared to 58% in regular snacks [21]. Additionally, studies report that low-protein baby cereals, bread, and milk substitutes contain three times the fat content of their regular equivalents [12]. Meat substitutes, pasta, rice, and soup products contain more fat than regular products. Low protein crackers, yogurt and bread contained 73%, 60% and 34% more saturated fatty acids (SFAs) compared with regular foods [19, 24]. The SFAs content of low protein bread and mixes were 2 fold higher than regular equivalents, this ratio was 1.1 and 1.5 fold for cheese substitutes and low protein pasta respectively [23].

#### Salt

Studies highlight that SLPFs often contain higher salt levels than regular protein-containing foods. In the UK, Wood et al. found that 25% of SLPFs had an elevated salt content, with the most significant differences observed in rusk, hazelnut spread, hot breakfast cereal, crisps, cake, potato pots, and pizza mix, where salt levels were 50% to 1050% higher than conventional products [21]. Similarly, Arslan et al. analyzed 148 SLPFs in Turkey and reported that all contained significantly more salt, on average 358.3% higher than regular foods. The largest disparities were found in bread, flour, pasta, and rice [19]. Another study further identified cheese, meat, and fish substitutes, particularly low-protein flour and pasta, as having substantially higher sodium content compared to regular foods [23].

#### Food additives

Food additives, known by their E numbers or E codes are widely used by the food industry in processing foods to improve texture, and flavor, and extend shelf life. They add more colour to enhance the appeal of foods [25]. Seventy seven per cent of SLPFs, contain fiber but predominantly in the form of hydrocolloids including methyl cellulose (MC) (E461), hydroxypropyl-methylcellulose (HPMC) (E464), guar gum (E412), carob/locust bean gum (E410), carrageenan (E407), xanthan gum (E415), and carboxymethyl methyl cellulose (CMC) (E466). Hydrocolloids are widely employed as food additives, functioning as emulsifiers, stabilizers, thickeners, and anti-staling agents in bakery applications. Their use is particularly important in gluten-free (GF) products and SLPFs, where they enhance the rheological, functional, and organoleptic properties of starch. Specifically, hydrocolloids improve elasticity, viscosity, gelation behavior, and gas retention, thereby contributing to product quality and consumer acceptability [26–34].

#### Micronutrients

Most SLPFs do not contain added micronutrients or trace elements. In a survey of 73 products, Peña et al. reported that 43 lacked any vitamin content or did not provide information on food labels. Furthermore, 48 products had no declared magnesium, zinc, or iron content [12]. Fortification of SLPFs with micronutrients is uncommon, as the majority of vitamins and minerals are typically supplied through protein substitutes, which are generally supplemented with essential micronutrients [12, 35].

### The nutritional contribution of SLPFs in patients with amino acid metabolism disorders

Studies indicate that SLPFs contribute significantly to the daily dietary intake of individuals with PKU and other AMDs requiring lifelong protein restriction. The mean reported daily consumption of SLPFs is 412 g/day for PKU patients and 346 g/day for other AMDs [36]. In Italy, Germany, and the UK, SLPFs account for 47%, 39%, and 33% of total daily energy intake, respectively [22, 37]. Most SLPFs contain 305–478 kcal/ 100 g [12].

Children with PKU typically consume a higher percentage of carbohydrate than healthy individuals, often with an elevated dietary glycemic index [37]. According to Couce et al., the mean daily carbohydrate intake for PKU patients was 282.59 ± 68.9 g, of which 57.042 ± 8.5 g came from protein substitutes [38]. Another study found that the combined total carbohydrate intake from protein substitutes, SLPFs, and regular foods averaged 294 g/day, with starch providing 174 g/day (59%) and sugar 119 g/day (40%) [22]. SLPFs contributed 40% (SD ± 10) of the total daily carbohydrate intake, with starch providing 51% (SD ± 18) and sugar 21% (SD ± 8). Among patients with PKU, low-protein bread and pasta were the largest contributors to carbohydrate intake, averaging 50 g/day and 37 g/day, respectively. Notably, carbohydrate and sugar intake from SLPFs was nearly twice as high as carbohydrate intake from protein substitutes [23]. Furthermore, a significant positive correlation was observed between carbohydrate consumption and intake of sugars, total fats, SFAs, monounsaturated fatty acids (MUFAs), and polyunsaturated fatty acids (PUFAs).

### Adverse effects of food ingredients added to SLPFs on human health

#### Gut microbiota dysbiosis and inflammatory markers induction

**Starch**: this is the primary component in almost all SLPFs due to its low protein content. However, excessive consumption of refined or simple carbohydrates has been associated with inflammation and negative effects on gut microbiota, potentially disrupting microbial balance and metabolic function [39, 40].

**Carrageenan**, a widely used thickening agent and food additive, is commonly incorporated into processed foods and SLPFs, including meat substitutes, desserts, and dairy‑based low‑protein alternatives. However, numerous clinical trials have raised concerns regarding its adverse health effects [41–44]. Research suggests that carrageenan may contribute to gut microbiota dysbiosis by significant inhibitory effect on growth of *Bifidobacterium* [45] and reducing *Faecalibacterium* populations while increasing *Bacteroides*, leading to pro-inflammatory microbiota gene expression [46–48]. Additionally, it has been shown to thin the protective outer mucus layer of the intestinal epithelium, potentially triggering inflammatory bowel disease (IBD) and allergic reactions [41, 42, 49]. In a cohort study comparing patients with IBD to healthy controls, children with IBD demonstrated significantly higher exposure to carrageenan than adults (0.33 ± 0.43 vs. 0.18 ± 0.32; *P* = .02) [50]. Furthermore, significant changes were observed indicating that carrageenan intake may induce calprotectin production and elevate IL‑6 levels [51]. These findings suggest that carrageenan exposure could exacerbate inflammatory pathways, particularly in pediatric IBD populations. Kappa carrageenan (κ-CGN) increases secretion of inflammatory cytokines particularly TNF-α and also IL-1β, IL-6 and IL-8 in a dose dependent manner. In a study by Jiang et al. the effects of three doses of κ-CGN were assessed on an in vitro co-culture system utilizing intestinal epithelial Caco-2-cell monolayer and phorbol myristrate acetate activated THP-1 macrophage cell. κ-CGN-induced severe damage to Caco-2 cell monolayers in the first 4 h of treatment with the highest dose of κ-CGN (10 µg/ml) [43]. Also, a positive correlation with CGN intake and Crohn’s disease in children was reported by Lee et al. [52].

**Carboxymethyl cellulose (CMC)** is commonly used in processed foods, but research has indicated its potential impact on gut microbiota. Studies have shown that CMC can lead to a significant reduction in *Lactobacillales*, including the *Streptococcus genus*, as well as depletion of *Faecalibacterium prausnitzii* and *Ruminococcus* [53].

The depletion of *Faecalibacterium prausnitzii* is particularly concerning, as this bacterium is one of the most abundant butyrate producers, known for its anti-inflammatory properties and its role as a gut health indicator. Previous studies have found a positive correlation between the loss of this microbiota and the development of IBD [54–56], and ulcerative colitis [57]. A review by Cao et al. found that expression of flagellin gene has positive correlation with intake of CMC in mice and in a model study that used a mucosal-simulated human intestinal microbiota ecosystem (M-Shime) [58, 59]. It also promotes colon carcinogenesis [58, 60].

Both CMC and carrageenan have been shown to alter gut microbiome composition, leading to intestinal inflammation by eroding the protective mucosal layer and inducing pro-inflammatory molecule expression [44, 46].

Maltodextrin, propylene glycol alginate, arabic gum, CMC, and locust bean gum have been linked to negative effects on microbiota gene expression and composition [46, 59]. Research suggests that these ingredients may contribute to microbial imbalances, influencing digestive function and inflammatory pathways. Additionally, locust bean gum has been associated with undesirable gastrointestinal effects in infants and toddlers consuming FSMPs and specialized formula. Notably, some of these formulas contained 10 times more locust bean gum than standard infant formula [61].

**Oil and fatty acids**: palm oil is one of the primary fat sources used in SLPFs, but its health implications raise concerns.

Diacetyl tartaric acid ester of mono-and diglycerides (DATEM), E472e increase *Bacteroides* and decrease *Faecalibacterium* [46]. 

A cohort study involving 104-139 participants investigated the impact of emulsifier intake on its association with type 2 diabetes risk. The study found a positive correlation between the intake of emulsifiers, E407 (carrageenan), E412 (guar gum), E414 (arabic gum), E415 (xanthan gum), and E472e and type 2 diabetes [62].

A high emulsifier intake is likely when consuming SLPFs given that they provide a high energy source in the diet of individuals with AMDs. However, accurately estimating emulsifier intake remains challenging, as manufacturers typically list their presence without specifying exact quantities.

**Monosodium glutamate** (MSG) is a widely used flavor enhancer responsible for creating the umami taste in processed foods. While it enhances flavor, studies in both humans and animals have linked MSG consumption to obesity and a positive correlation with hepatotoxicity, neurotoxicity, and oxidative stress [63, 64]. A systematic review by Ahangari et al. found that gut dysbiosis can occur with consistent MSG intake, as excessive consumption increases *Bacteroidetes* abundance, which has been associated with a heightened risk of cardiovascular disease [65]. Additionally, MSG has the potential to accumulate trimethylamine oxide (TMAO) in the serum, a compound linked to metabolic and cardiovascular health risks, while simultaneously reducing *Akkermansia muciniphila* (A. *muciniphila*), a beneficial gut microbiota species known for its anti-inflammatory properties [65–67]. A reduced level of *A.muciniphila* has been reported in patients with IBD and ulcerative colitis [67, 68]. Also an inverse correlation between *A.muciniphila* and serum TMAO is observed in patients with colon cancer [69]. Protective effects of *A. muciniphila* on gut health and immune response were shown in previous studies. It can improve gut barrier integrity and permeability by antimicrobial and anti-inflammatory effects particularly in patients with IBD [70]. *A. muciniphila*, is a promising next-generation probiotic candidate. When administered in its pasteurized form as a postbiotic, it has been associated with a significant reduction in circulating levels of TMAO [71].

**Artificial food dyes**: the widespread use of artificial food colorants, such as Allura Red (Red 40) and Sunset Yellow, serves to enhance the visual appeal of processed foods. Recent human studies have reinforced concerns regarding the impact of artificial food dyes on gut microbiota dysbiosis and inflammation, emphasizing their potential role in disrupting digestive health [72, 73]. Artificial food colours have also been associated with the development of attention deficit hyperactivity disorder (ADHD), irritability, allergic reactions, and they have carcinogenic potential. Notably, dyes such as Quinoline Yellow, Tartrazine, Red 40, and Blue Dye have been implicated in these adverse health effects [74–76].

#### Metabolic syndrome and cardiovascular disease risk factors

Food additive consumption has been linked to an increased risk of metabolic syndrome indicators, including abdominal obesity, impaired glucose tolerance, dyslipidemia, hypertension, insulin resistance, and type 2 diabetes. These associations are thought to be mediated largely through the impact of additives on gut microbiota dysbiosis [62, 77, 78].

In a double‑blind, placebo‑controlled study, overweight individuals who received oral carrageenan (250 mg twice daily for two weeks) demonstrated evidence of insulin resistance [79]. Furthermore, higher intake of cellulose derivatives (E460–466), as well as monoglycerides and diglycerides of fatty acids (E471–E472), has been associated with an elevated risk of cardiovascular disease (CVD) [80]. A cohort study of 104139 participants identified monoglycerides and diglycerides of fatty acids as a major source of total emulsifier intake, second only to modified starches [62]. Additionally, an animal study by Kyaw et al. showed that MSG consumption reduces TMAO urine excretion, increasing serum TMAO concentration [66], which correlates with fat deposition in blood vessels, potentially heightening the risk of atherosclerosis and CVD [81–85].

These findings suggest a potential link between CVD risk and the frequent consumption of SLPFs that are low in protein but high in calories, fat, and additives. A recent study by Santos et al. (2025) supports this hypothesis, reporting an increased CVD risk in adults with PKU, possibly due to obesity, dyslipidemia, kidney dysfunction, gut dysbiosis, and oxidative stress [86].

**Fig. 1:**
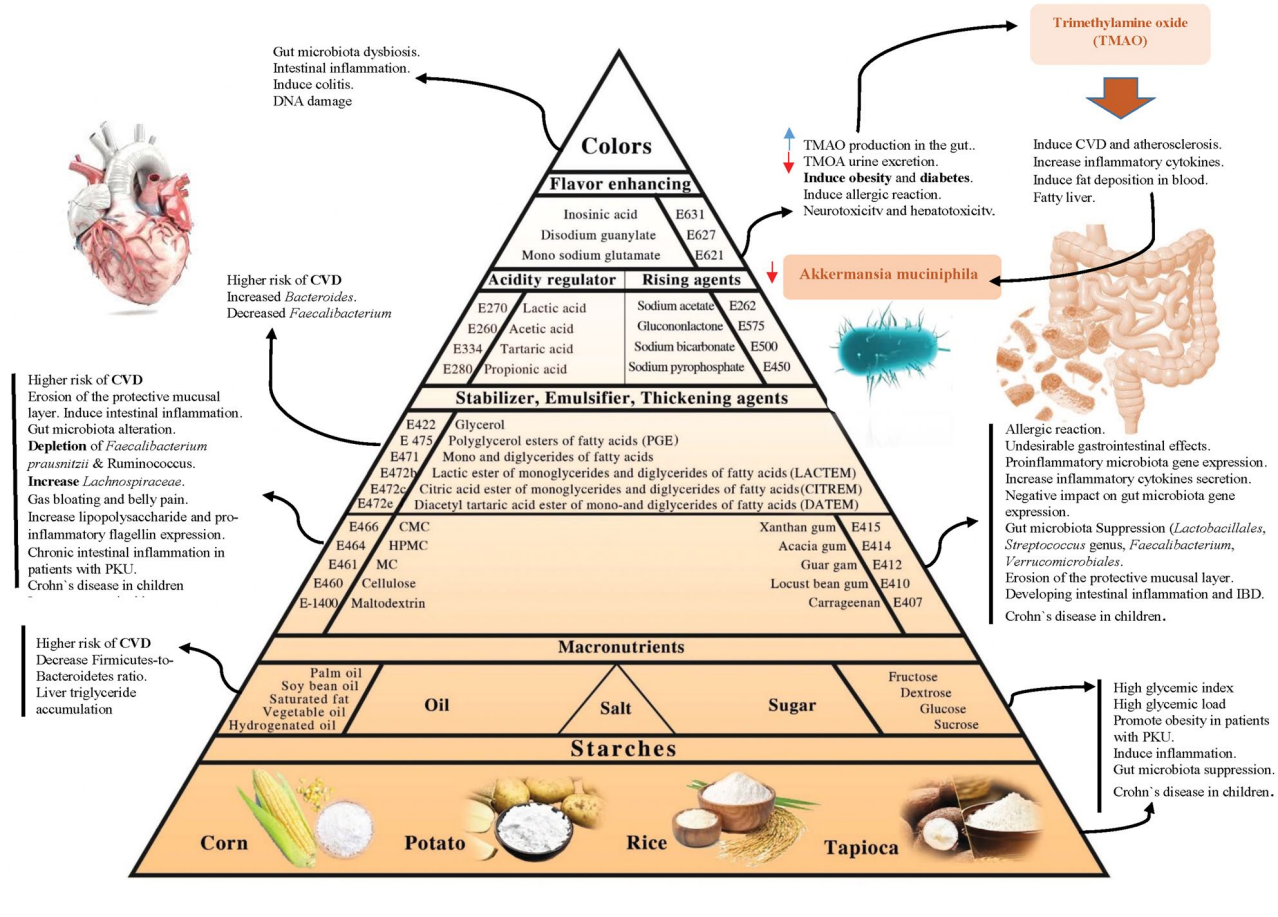
Common ingredients added to special low protein foods and their adverse effects on humans health

While studies focus on the effects of food additives in healthy individuals, patients with AMD rely on SLPFs daily and commonly have a high exposure. A pyramid model (Fig. 1) provides a schematic overview of the principal ingredients used in SLPFs and highlights the adverse effects associated with common additives.

### How SLPFs May influence the health of patients with amino acid metabolism disorders

The main ingredients added to SLPFs and their potential impact on health in patients with AMD’s are given in Fig. 1.

#### Gut microbiota alteration reported in patients with amino acid metabolism disorders

A recent study by Garcia et al. showed that the dietary pattern of patients with AMD’s could be influenced by the nutritional composition of the SLPFs [23]. Depletion of the two main butyrate-producing species of microbiota (*Faecalibacterium* and *Roseburia* from the *Firmicutes* genera) have been reported in children with PKU and patients with PA [87–89] resulting in significant reduction in SCFA production, with butyrate depletion observed in both disorders compared with the healthy population. Verduci et al. noted that the high glycemic index and glycemic load of a Phe restricted diet affects the substrate of microbial fermentation [88, 89]. Therefore, reliance on SLPFs may influence SCFA production. This hypothesis is supported by findings from Bassanini et al., who reported a negative correlation between glycemic index and the depletion of *Faecalibacterium*, a Firmicutes genus known for its butyrate-producing and anti-inflammatory properties, in patients with PKU [87]. Similarly, a study has shown that individuals with glycogen storage disease type 1, who maintain a lifelong cornstarch-rich diet, experience a significant reduction in *Faecalibacterium* and *Ruminococcus* populations [90]. Research by Pinheiro et al. also showed marked differences in the *Firmicutes phylum* between PKU patients and healthy controls, with notable depletions in *Clostridiaceae*,* Ruminococcus*, and *Lachnospiraceae* in individuals with PKU [91]. Additionally, *Veillonellaceae* depletion has been observed in children with PKU [87].

A meta-analysis by Ubaldi et al. (2023) found that a Phe-restricted diet tended to reduce the *Bacillota/Bacteroides* ratio, a key indicator of gut health [92]. This ratio has been associated with exercise intensity, as reported in a systematic review by Ghaffar et al. (2024), suggesting its relevance in metabolic adaptation [93]. Additionally, research has shown that individuals with IBD tend to have a lower *Bacillota/Bacteroides* ratio, highlighting its potential connection to gut microbiota balance and inflammatory conditions.

#### Prevalence of cardiovascular disease risk factors in patients with amino acid metabolism disorders

Dyslipidemia is a major marker of CVD prevalence [94], with elevated inflammatory cytokines and C-reactive protein (CRP) contributing to CVD progression by promoting vascular dysfunction and systemic inflammation [95].

A study by Azabdaftari et al. found that adults with PKU (ages 18–47) had higher total cholesterol and triglyceride levels, together with lower high-density lipoprotein (HDL) compared to healthy individuals. Additionally, patients with PKU showed increased inflammatory markers, endothelial dysfunction, vascular stiffness, and higher systolic and diastolic blood pressure, suggesting a heightened cardiovascular risk associated with their dietary restrictions [96]. Research has consistently linked arterial stiffness in adult patients with PKU to metabolic and cardiovascular concerns [97]. Hermida et al. observed this effect in individuals aged 6 to 50 years [98], while studies on obesity in children and adolescents with PKU (ages 4–15) reported lower plasma HDL levels and higher triglyceride (TG) concentrations [99].

Similarly, studies by Fernandez et al. and Htun et al. found that adults with PKU exhibited lipoprotein profiles comparable to those in healthy individuals, reinforcing the association between PKU and altered lipid metabolism [100, 101]. The recent findings by Santos et al. (2025) provide an in-depth analysis of the prevalence of cardiovascular disorders in individuals with PKU, highlighting multiple metabolic risk factors [86]. 

#### Metabolic syndrome prevalence and insulin resistance in patients with amino acid metabolism disorders

SLPFs tend to have a high glycemic index due to their nutritional composition, particularly the frequent use of maize/corn starch as the primary carbohydrate source [21]. Additionally, some GF foods, often low in protein, may be incorporated into protein-restricted diets [102, 103]. Many cereal-based GF products contain large amounts of corn starch and sugar, with studies showing that 56% of 629 GF cereal-based products contained sucrose [104]. Research by Couce et al. found that energy intake was higher in patients with classical PKU compared to those with hyperphenylalaninemia (HPA). This increased intake was correlated with basal insulin levels, homeostasis model assessment insulin resistance (HOMA-IR), and body mass index (BMI) [38, 99].

Insulin resistance (IR) has been positively correlated with central obesity in patients with PKU [99, 105]. Furthermore, IR has been reported in PKU due to the high carbohydrate intake from SLPFs [21, 37, 38], which may contribute to the development of CVD [106].

#### A practical approach to improving the nutritional integrity of special low protein foods

**Fig. 2:**
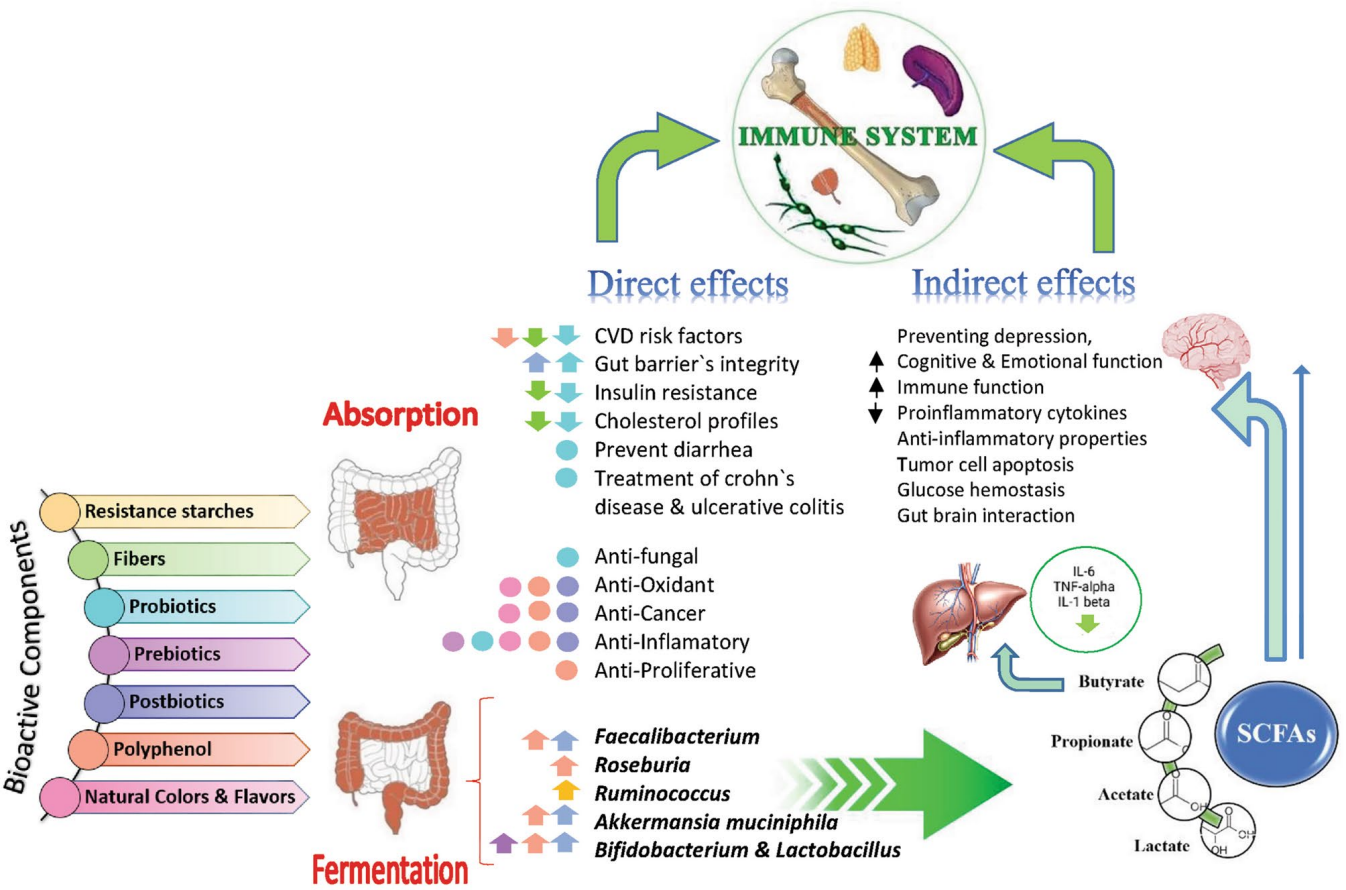
Health benefits of adding bioactive ingredients in special low protein foods

The nutritional integrity of SLPFs can be advanced through the strategic incorporation of natural ingredients and bioactive components. The health benefits of adding bioactive ingredients to SLPFs are given in Fig. 2.

Practical strategies for adding natural foods and bioactive components to SLPFs to improve their nutritional quality are given in Table 3.

**Table 3:** Strategies for adding natural ingredients and bioactive components to SLPFs

Bioactive components	Sources	Strategy	Target low protein foods
**Resistance starch (RS)**	Green banana flour, taro, cassava, potato, yam, Jerusalem artichoke and bread fruit	**Fortification**: Add milled powder	Crackers, bars, biscuits, cake, cereals, pasta, noodle, soup, breads
**Polyphenols**	Berry and grape anthocyanins, purple carrot anthocyanins and chlorogenic acids, green tea catechins, turmeric curcumin, grape skin and leaf gallic acid	**Fortification with extracts**: Incorporate concentrated plant extracts	Baked products, beverages snack bars.
	Beetroot, purple carrot, hibiscus	**Natural coloring/flavoring**: Use polyphenol‑rich powders, replacing artificial additives	
		**Encapsulation**: Apply microencapsulation to stabilize polyphenols during processing and improve bioavailability	Baked goods Meat replacer beverages, snack bars.
**Prebiotics**	Blue Agave, yacon root, bread fruit, Jerusalem artichoke, chicory root, asparagus, bananas. Aquafaba	**Fiber enrichment**: Add: fructo‑oligosaccharides (FOS) or galacto‑oligosaccharides (GOS)	Dairy substitutes flour, breads/cereals. Protein substitutes (including infant formulas)
		Blend with resistance starch to enhance fermentation and SCFA production	Flour, breads, cereals
**Probiotics**	*Lactobacillus* and *Bifidobacterium*	**Fortification** with shelf‑stable powders	Dairy free yogurt, dairy substitutes
		Incorporate freeze‑dried probiotic cultures Blend with prebiotic (symbiotic) to maximize gut microbiota modulation	Low-protein drink mixes or snack bars.
**Postbiotics**	Akkermansia muciniphila metabolites, SCFAs, polysaccharides, nisin, and conjugated linoleic acid (CLA) derived from bacteria	**Functional fortification**: add postbiotic metabolites, Utilizing stabilized postbiotic compounds	Low-protein supplements or ready-to-eat foods
**Phytochemicals**	Carotenoids in pumpkin, carrots, tomatoes; carotenoid enriched oils, glucosinolates extract, polyphenols in berries	**Replace artificial flavors/colors**	Baked products
**Natural color**	Purple sweet potatoes, purple carrot, purple cabbage, spinach powder, elderberry extract, blueberry, pumpkin and mango powder, beet root betalain, grape skin anthocyanin	**Fortification with extracts**: incorporate fresh or concentrated fruits and vegetable extracts	Meat replacer, pasta, bread, biscuits, cereals, muffins, bars, ice cream, jellies and gummies
**Natural flavor**	Vanilla, rose water, saffron, cardamom, nutmeg, thyme, mint, citrus, tangerine flowers and coconut and jackfruit essences. Fruit puree: strawberry, mango, banana, apple, jackfruit	**Fortification with natural flavours**:	Baked products, pasta, rice, meat replacer, burger, sausages, snacks, jellies, drink mixes

**Resistant starch** plays a vital role in gut microbiota fermentation, particularly benefiting *Ruminococcus bromii*, a genus strongly linked to high-yield butyrate production [107–110]. Butyrate is a key SCFA that supports colonic health, modulates inflammation, and contributes to metabolic homeostasis. In addition, dietary fibers, together with prebiotic and probiotic supplements, help regulate gut microbiota composition and enhance SCFA levels.

Foods such as unripe bananas are naturally rich in resistant starch and contain minimal protein. Cooking followed by cooling induces retrogradation, increasing resistant starch content. Potato starch is inherently low in protein, making it highly compatible with low‑protein dietary products. Tapioca starch is widely used in low‑protein formulations due to its negligible protein content and can be processed to increase its resistant starch content.

**Probiotics** have demonstrated a wide range of health benefits across diverse patient populations. Evidence supports their role in the treatment of Crohn’s disease and ulcerative colitis, in reducing the severity of rotavirus diarrhea in infants and children, and in lowering the risk of invasive fungal infections among preterm neonates. Probiotics have also been shown to improve IR, inflammatory markers, cholesterol profiles, minimize CVD risk factors, and prevent eczema in infants and children [111–116]. Shelf‑stable powders containing freeze‑dried probiotic strains may be incorporated into low protein drink mixes or snack bars.

**Prebiotics** are food ingredients that resist digestion by gastric acidity and are not absorbed in the gastrointestinal tract [117]. Galacto‑oligosaccharides (GOS) and fructo‑oligosaccharides (FOS) are among the most studied prebiotics, with strong potential to selectively support the growth of beneficial gut microbiota [118–121].

Clinical evidence demonstrates that infant formulas supplemented with GOS/FOS mixtures positively stimulate the growth of *Bifidobacteria* and *Lactobacilli* [122–125]. Similar findings were reported by MacDonald et al. in infants with PKU, where those receiving a protein substitute enriched with a 9:1 ratio of short‑chain galacto‑oligosaccharides (scGOS) and long‑chain fructo‑oligosaccharides (lcFOS) showed enhanced growth of beneficial microbiota compared to infants receiving protein substitutes without prebiotics [126].

**Postbiotics** derived from *Akkermansia muciniphila* may help reduce trimethylamine‑N‑oxide (TMAO) production in the gut while simultaneously promoting the growth of key microbiota that are often suppressed in patients with PKU. These include *Faecalibacterium*,* Bifidobacterium*, and *Lactobacillus*, all of which play important roles in maintaining gut health and immune regulation [71]. Postbiotics can be integrated into supplements or ready‑to‑eat foods to deliver functional benefits without requiring live organisms. Heat‑stable compounds such as SCFAs and polysaccharides are particularly suitable for incorporation, as they withstand processing conditions while supporting gut barrier integrity and anti‑inflammatory pathways.

**Phytochemicals** are bioactive compounds that play a key role in supporting human health and in the prevention of a wide range of diseases, particularly CVD and cancer. They are broadly classified into five major subgroups: polyphenols, carotenoids, flavonoids, alkaloids, and glucosinolates [127]. Each subgroup encompasses diverse compounds with distinct mechanisms of action, including antioxidant activity, modulation of signaling pathways, and regulation of inflammatory responses. Incorporating phytochemicals into the formulation of SLPFs may enhance their nutritional integrity, offering protective health benefits and supporting long‑term disease prevention for patients reliant on lifelong protein‑restricted diets. In SLPFs artificial flavors and colors can be replaced with phytochemical‑rich natural sources, such as carotenoid‑containing pumpkin or polyphenol‑rich berries. Carotenoid‑enriched oils and glucosinolates extracts may be used in spreads and cooking substitutes, while dried vegetable and fruit powders can be incorporated into low protein pasta, bread, and snack formulations.

**Polyphenols** play a pivotal role in strengthening the intestinal barrier by promoting the growth of beneficial bacteria such as *Lactobacillus* and *Bifidobacterium*. They also enhance butyrate‑producing bacteria, including *Faecalibacterium prausnitzii* and *Roseburia*, which contribute to anti‑inflammatory processes. In addition, polyphenols encourage the presence of *Akkermansia muciniphila*, *Bacteroides*,* Prevotella*, and *Bacteroides vulgatus*, while decreasing the *Firmicutes/Bacteroidetes* ratio, a shift associated with improved gut health [126]. In addition to their microbiota‑modulating effects, polyphenols exert diverse biological activities.

A recent review by Lou et al. highlighted the broad biological activities of specific polyphenols, including anthocyanins, apigenin, catechins, curcumin, and ellagitannins. These compounds demonstrated anti‑inflammatory, anti‑cancer, antioxidant, and anti‑proliferative effects, highlighting their potential as functional bioactive agents in dietary interventions [128]. Fortification strategies may include concentrated plant extracts such as berry anthocyanins, green tea catechins, or turmeric curcumin incorporated into baked goods, beverages, and snack bars. Polyphenol‑rich powders such as beetroot or hibiscus may also serve as natural colorants and flavor enhancers, replacing artificial additives. To ensure stability and bioavailability, microencapsulation techniques can be employed during processing.

**Natural colors** are generally considered safe for incorporation into food products. They contain bioflavonoids, antioxidants, and other bioactive compounds with nutritional and medicinal qualities [129, 130]. Common natural dyes include anthocyanins (E163), carotenes (E160a), riboflavin (E101), betalains (E162), and chlorophylls (E140) [131]. The health benefits of these compounds are well established: anthocyanins, betalains, and carotenoids exhibit anti‑cancer, antioxidant, and anti‑inflammatory properties [132–137]. In addition, natural colors have been shown to modulate gut microbiota by increasing the abundance of beneficial bacteria such as *Bifidobacterium*,* Lactobacillus*,* Bacteroidetes*,* Akkermansia*, and *Roseburia* [138]. Natural colors derived from foods such as purple sweet potato, blueberry, carrot, pumpkin, beetroot, and elderberry enhance both the sensory appeal and nutritional value of SLPFs.

**Natural flavors** are increasingly recognized as functional ingredients due to their diverse bioactive properties. They contain compounds with antioxidant, anti‑inflammatory, anticancer, and antimicrobial activities, which collectively contribute to immune support and may offer therapeutic potential in conditions such as depression, cancer, and cognitive decline, as well as in maintaining gastrointestinal health [139]. Natural flavors can enhance the sensory quality of SLPFs, improving palatability and their acceptability. Examples include vanilla, fruit essences, cardamom, saffron, nutmeg, and citrus extracts, all of which provide distinctive aromatic and flavor profiles while contributing functional health benefits.

## Discussion

SLPFs primarily consist of starch, oil, and sugar (SOS) as energy sources, and are deliberately designed to be low in protein. GF products share similar characteristics, being dense in SOS ingredients but low in protein [104]. Both SLPFs and GF foods tend to have a high glycemic index and offer limited nutritional benefits [30, 37, 38]. Many additives, such as maltodextrin, MCs, xanthan gum, carrageenan, guar gum, Arabic gum, locust bean gum, HPMC, and CMC, are commonly included to enhance texture rather than add nutritional value [12, 21, 33, 34, 104]. However, research suggests that these additives may contribute to inflammation, gut microbiota imbalances, metabolic syndrome, and increased risks of type 2 diabetes and CVD [50, 62, 67, 80]. Gut microbiota development is shaped by multiple factors, including genetics, diet, lifestyle, medications, and environmental influences, especially during the first three years of life, when significant changes occur [140, 141]. Dietary patterns play a crucial role in microbiota composition and are influenced by cultural habits and nutrient sources [142, 143]. Evidence suggests that frequent consumption of SLPFs may suppress beneficial gut bacteria, and this is likely to have negative impact on individuals with PKU and other AMDs [87–89, 91, 142, 144, 145]. In addition, treatment strategies, medications, and genetic predisposition also impact microbiota health in patients with these conditions [146].

Gut microbiota is a diverse community of microorganisms residing in the human gastrointestinal tract, particularly in the colon, where it plays a critical role in overall health [147–149]. Extensive research has demonstrated its protective effects against various conditions, including obesity [150–154], diabetes [150, 155–157], ulcerative colitis, Crohn’s disease and IBD [158, 159], cancer [160–163], neuropsychiatric disorders [164, 165], neurodegenerative disease [166], and musculoskeletal diseases [167]. One of its essential functions is the production of SCFAs through the fermentation of dietary fiber, resistant starch, and oligosaccharides, mainly FOS and GOS [120]. SCFAs contribute to numerous biological processes, including tumor cell apoptosis, glucose hemostasis and gut-brain interactions [168–170]. Butyrate, in particular, helps regulate inflammation by inhibiting the production of proinflammatory cytokines [171] such as IL-1, IL-6, and TNF-α. It also suppresses TNF-α and IL-6 by blocking NF-kB activation in liver Kupffer cells [172]. The protective effects of SCFAs extend to neurological conditions like depression and Alzheimer’s disease [173], as well as cognitive, emotional, and immune functions [168, 174, 175]. The findings from this review highlight that the primary constituents of these SLPFs may exert adverse physiological effects, notably through the induction of proinflammatory cytokines and the depletion of beneficial butyrate-producing gut microbiota.

SLPFs are a fundamental component of dietary therapy for individuals with AMDs. However, consistent and prolonged reliance on these products raises notable concerns regarding both immediate and long-term health outcomes. Regular intake has been associated with an elevated risk of developing metabolic syndrome, as classified by the International Diabetes Federation [176]. Vulnerable subpopulations, particularly toddlers, children, and adolescents, may be especially susceptible to the potential adverse effects of cumulative exposure to food additives, commonly found in SLPFs.

To improve the nutritional quality of SLPFs, establishing clear dietary standards is essential [12]. Additionally, mandatory labeling should be required, ensuring full transparency regarding nutritional composition so health professionals and patients can make well-informed dietary decisions, an issue emphasized in previous research [12, 19, 21]. Future investigations should prioritize the systematic evaluation of additive content of SLPFs, with the aim of establishing precise estimates of daily intake across relevant age groups. Such assessments are critical in determining potential correlations between chronic additive exposure and the emergence of metabolic comorbidities, thereby informing evidence-based guidelines for safe formulation and regulatory oversight.

Improving the nutritional profile of SLPFs requires a deliberate integration of functional components that support both metabolic control and broader health outcomes. The inclusion of resistant carbohydrates, probiotic, prebiotic and postbiotic sources, polyphenols, phytochemicals, natural colors and flavors, as well as both soluble and insoluble fibers, are essential. These elements contribute not only to enhanced gut health and microbiome diversity but also play a preventive role in mitigating long-term comorbidities such as gastrointestinal dysfunction, inflammation, and metabolic syndrome. Their incorporation should be guided by evidence-based formulation principles, with attention to palatability, age-appropriateness, and the dietary restrictions of individuals with AMDs.

### Limitations

We acknowledge several limitations in this narrative review. First, the absence of a structured, reproducible search and selection process restricts both the comprehensiveness of study identification and the transparency of inclusion criteria. As such, relevant studies may have been overlooked, and the potential for selection bias cannot be excluded. Our intention, however, was to provide a broad synthesis of available evidence and contextual insights, rather than a systematic meta-analysis. This approach allowed us to highlight emerging themes, translational opportunities, and practical applications, though it necessarily reduces reproducibility and generalizability. Furthermore, the heterogeneity of study designs, and outcome measures across the literature complicates direct comparison and synthesis.

## Conclusion

This narrative review highlights the urgent need to improve the nutritional quality of SLPFs to better support individuals adhering to lifelong protein-restricted diets. Given patients’ sustained reliance on these products, it is imperative to advance the development of SLPFs with optimised nutrient profiles, enhanced palatability, and improved accessibility. Strengthening these dimensions will be instrumental in supporting long-term health outcomes and maintaining dietary adherence. Further research is essential to drive innovation and address persistent nutritional deficiencies inherent in current formulations.

## Data Availability

Not applicable.

## Abbreviations

ADHD: Attention deficit hyperactivity disorder
AMD: Amino acid metabolism disorders
CMC: Carboxymethyl cellulose
CRP: C - reactive protein
CVD: Cardiovascular disease
DATEM: Diacetyl tartaric acid ester of mono-and diglycerides
FOS: Fructooligosaccharides
FSMP: Foods for special medical purpose
GF: Gluten free
GI: Glycemic index
GOS: Galactooligosaccharides
HCU: Homocystinuria
HDL: High density lipoprotein
HOMA-IR: Homeostasis model assessment insulin resistance
HPA: Hyperphenylalaninemia
HPMC: Hydroxypropyl-methylcellulose
IBD: Inflammatory bowel disease
IMD: Inherited metabolic disorde
κ-CGN: Kappa carrageenan
MC: Methyl cellulose
MGG: Monosodium glutamate
MSUD: Maple syrup urine disease
MUFAs: Monounsaturated fatty acids
PA: Propionic acidemia
PKU: Phenylketonuria
PUFAs: Polyunsaturated fatty acids
SCFAs: Short chain fatty acids
SFAs: Saturated fatty acids
SLPFs: Special low-protein foods
TG: Triglyceride
TMAO: Trimethylamine oxide
TYR: Tyrosinemia
UCD: Urea cycle disorders
